# Publisher Correction: Unified framework for laser-induced transient bubble dynamics within microchannels

**DOI:** 10.1038/s41598-024-72091-x

**Published:** 2024-09-11

**Authors:** Nagaraj Nagalingam, Vikram Korede, Daniel Irimia, Jerry Westerweel, Johan T. Padding, Remco Hartkamp, Hüseyin Burak Eral

**Affiliations:** Process and Energy Department, Delft University of Technology, Leeghwaterstraat 39, 2628 CB Delft, Netherlands

Correction to: *Scientific Reports* 10.1038/s41598-024-68971-x, published online 13 August 2024

In the original version of this Article a previous rendition of Figure 2B, Figure 4 and Figure 5D was published. The original Figure 2, 4 and 5 and accompanying legends appear below.

**Figure 2:**
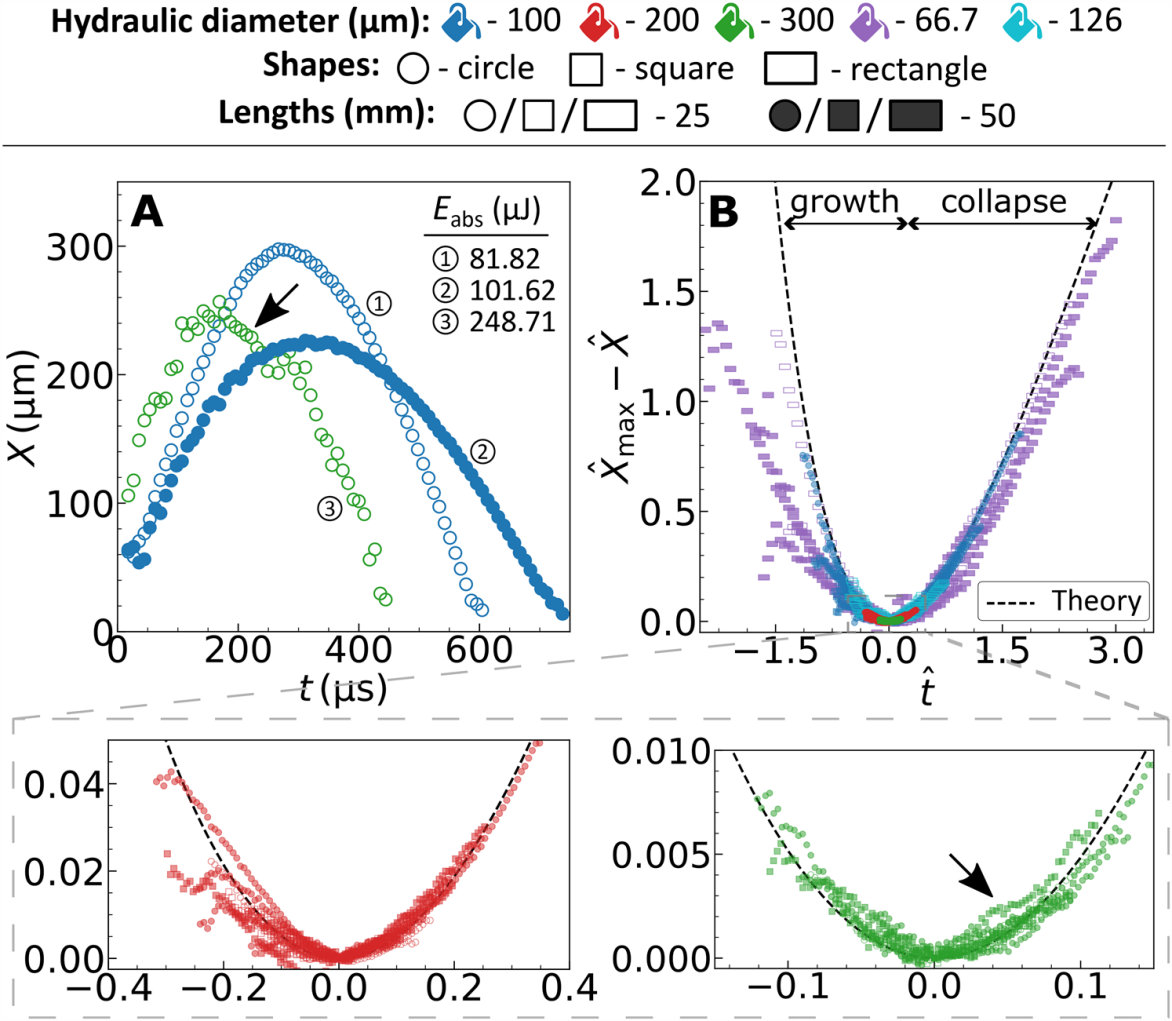
(**A**) Representative bubble dynamics for different channel geometries. (**B**) Universal motion of bubbles within channels with different size, shape and length. The dashed line represents the developed theory, Eq. (2). The marker colors represent the hydraulic diameters (*d*_h_), the shapes represent the cross-section and the facecolor represent the lengths (*L*). The graphical marker symbols and colors established here are followed throughout this article. The black arrow represents the region of deviation(s) from the expected dynamics.

**Figure 4:**
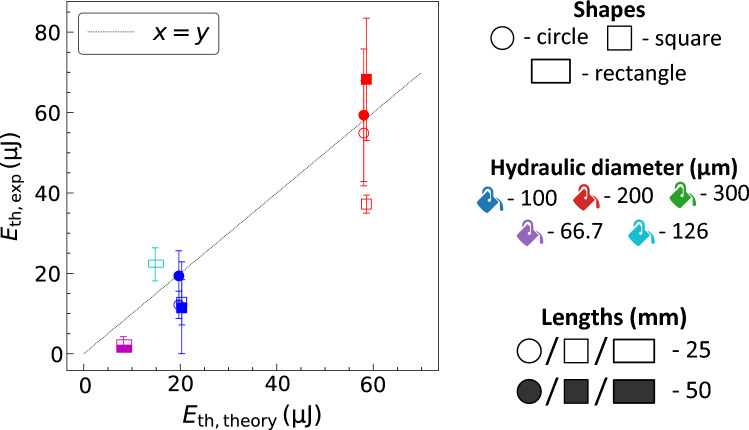
The threshold laser energy absorbed for bubble formation estimated from experiments (*E*_th,exp_) against theory (*E*_th,theory_) presented in Eq. (5).

**Figure 5:**
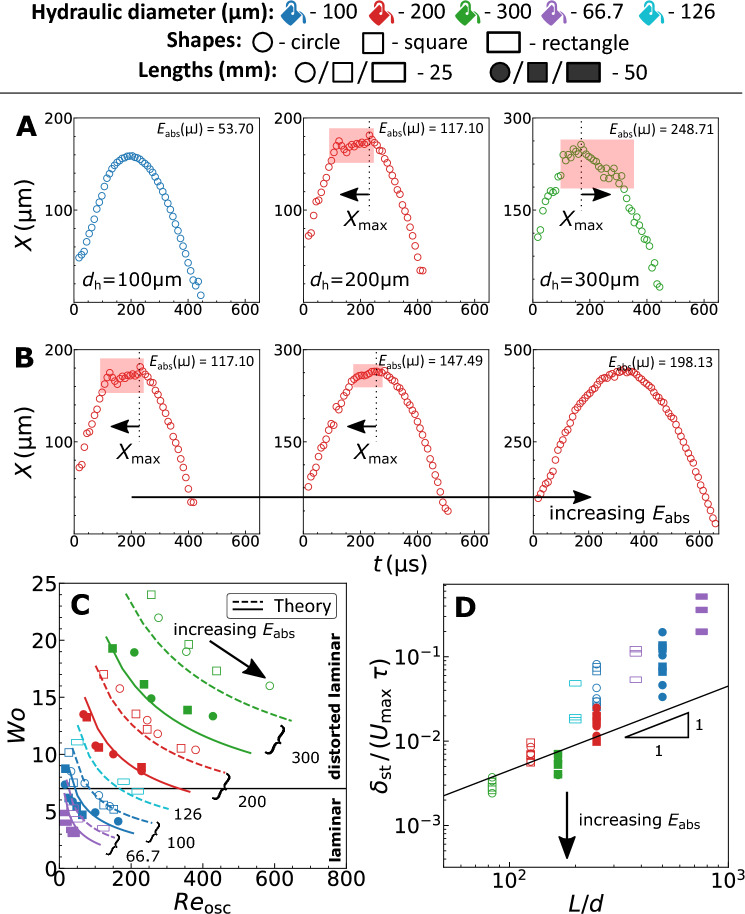
(**A,B**) Representative dynamic bubble size curves illustrating the emergence of instabilities. The zones of the instabilities are highlighted using a shaded rectangular area. The arrows represent if the instabilities occur before or after *X*_max_. (**A**) Illustrates the experimental data for different *d*_h_ with similar oscillation time. The instabilities emerge with increasing *d*_h_. (**B**) Illustrates the data for *d*_h_ = 200 µm with increasing laser energies. The instabilities disappear with increasing *E*_abs_. (**C**) Flow stability diagram with the transition line at *W*_o_ = 734. The markers represent the experiments and the lines represent the analytical estimate. The numbers correspond to the channel hydraulic diameters (in µm) with the dashed and solid lines representing the channel lengths L = 25 and 50 mm, respectively. (**D**) The dimensionless convective timescale against the *L*/*d*_h_ aspect ratio. The partition line is a linear relation between the *x* and *y* axes with 45 × 10^−6^ as the slope and the origin as the intercept.

The original Article has been corrected.

