# Supplementary material for: Unified framework for laser-induced transient bubble dynamics within microchannels
Source: Sci Rep. 2024 Aug 13;14:18763. doi: 10.1038/s41598-024-68971-x (PMC11322490; doi:10.1038/s41598-024-68971-x)
Supplement: Supplementary file 1 — Supplementary Information. [file 41598_2024_68971_MOESM1_ESM.pdf]

Unified Framework for Laser-induced Transient Bubble Dynamics within Microchannels

Nagaraj Nagalingam,[1] Vikram Korede,[1] Daniel Irimia,[1] Jerry Westerweel,[1]
Johan T. Padding,[1] Remco Hartkamp,[1] and Hüseyin Burak Eral[1,*]

[1] *Process & Energy Department, Delft University of Technology, Leeghwaterstraat 39, 2628 CB Delft, Netherlands*

CONTENTS

SI. Dynamic bubble size, bubble lifetime and bubble energy S1

SII. Laser energy absorbed by the liquid and threshold energy for bubble formation S2

SIII. Analytical solution to oscillating flow with sinusoidal pressure gradient S3

References S11

SI. DYNAMIC BUBBLE SIZE, BUBBLE LIFETIME AND BUBBLE ENERGY

Determining $\boldsymbol{X}$. Fig.S1 illustrates the image processing technique adapted to determine the bubble size. To eliminate the effects of curvatures on the estimation of X, we first calculate the bubble volume. The total volume of a vapor bubble, $V_{\mathrm{b}} = V_{\mathrm{cs}} + V_{\mathrm{cur},1} + V_{\mathrm{cur},2}$, can be expressed using the summation of the portions as expressed in Fig. S1. Where V_{cs} is the cross-section volume and V_{cur} is the curvature volume. The effective bubble length incorporating the curvature effects over the bubble ends is $X = V_{\mathrm{b}}/A$.

The volume $V_{\mathrm{cs}} = A(X_{12} - h_1 - h_2)$, where A is the cross-section area of the channel. $A = \pi d_{\mathrm{h}}^2/4$ for circular cross-section, and $A = a\,b$ for square/rectangular cross-section. d_{h} is the channel's hydraulic diameter and, a and b are the cross-section's edge lengths with $b \leq a$. The calculation of volume V_{cur} requires the consideration of the curvature.

Circular cross-section

The bubble volume is

$$V_{\mathrm{b}} = A\,(X_{12} - h_1 - h_2) + \sum_{i=1}^{2} \pi h_i\,(3d_{\mathrm{h}}^2/4 + h_i^2)/6, \tag{S1}$$

where,

$$h_i = R_i\,(1 - \cos(\theta_i)) \quad \text{and} \quad \theta_i = \sin^{-1}(d_{\mathrm{h}}/(2R_i)). \tag{S2}$$

Refer to Fig.S1 for all the other notations discussed in the section.

Square/Rectangle

The volumetric flow rate due to a curvature can be expressed as $\dot{V}_{\mathrm{cur}} = AU_{\mathrm{mean,cur}}$, where $U_{\mathrm{mean,cur}}$ is the average velocity. The average velocity for a rectangular channel is calculated as [1],

$$U_{\mathrm{mean,cur}} = \frac{a^2 \Delta p}{4 l_{\mathrm{L}} \mu_{\mathrm{L}}} \frac{\sin(\alpha_n)\beta_n}{AR\,\alpha_n^3} \left[AR - \frac{\tanh(\alpha_n AR)}{\alpha_n}\right]. \tag{S3}$$

Where $\alpha_n = \left(n - \frac{1}{2}\right)\pi$, $\beta_n = \frac{2(-1)^{n+1}}{\alpha_n}$ and $AR = b/a$. Furthermore, the velocity at the channel axis ($r = 0$) is [1],

$$U_{r=0} = \frac{a^2 \Delta p}{4 l_{\mathrm{L}} \mu_{\mathrm{L}}} \sum_{n=1}^{\infty} \frac{\beta_n}{\alpha_n^2} \left[1 - \frac{1}{\cosh(\alpha_n AR)}\right]. \tag{S4}$$

For square channels, $AR = 1$, since $a = b$. Furthermore, Eqs. S3 and S4 can be solved to obtain the velocity ratio, $U_{\mathrm{ratio}} = U_{\mathrm{mean,cur}}/U_{r=0} = 0.477$, which is independent of the channel's hydraulic diameter or the cross-sectional edge lengths. Thus $\dot{V}_{\mathrm{cur}}$ can be calculated as, $\dot{V}_{\mathrm{cur}} = 0.477A\,U_{r=0}$. Since $a = b = d_{\mathrm{h}}$, the cross-section area is $A = d_{\mathrm{h}}^2$. Analogous to $\dot{V}_{\mathrm{cur}}$, the V_{cur} is calculated by substituting $U_{\mathrm{r}=0}$ with h_i. We get, $V_{\mathrm{cur,i}} = 0.477\,h_i d_{\mathrm{h}}^2$. Thus for square channels,

$$V_{\mathrm{b}} = A\,(X_{12} - h_1 - h_2) + \sum_{i=1}^{2} 0.477\,h_i d_{\mathrm{h}}^2. \tag{S5}$$

In this work, we employ two rectangular channels with $AR = 0.5$ and 0.2667 for 50×100 (in µm) and 80×300 (in µm) channels, respectively. Below we present the results for rectangular channels similar to square:

$$V_{\mathrm{b}} = A\,(X_{12} - h_1 - h_2) + \sum_{i=1}^{2} 0.502\,h_i a\,b \quad \text{for } d_{\mathrm{h}} = 66.7\,\mu\mathrm{m}, \text{ and} \tag{S6}$$

$$V_{\mathrm{b}} = A\,(X_{12} - h_1 - h_2) + \sum_{i=1}^{2} 0.558\,h_i a\,b \quad \text{for } d_{\mathrm{h}} = 126\,\mu\mathrm{m}. \tag{S7}$$

Bubble lifetime and energy. The non-dimensionalized plots (unscaled) for dynamic bubble size are represented in Figs. S2-S4. Similarly, the non-dimensionalized plots (unscaled) for bubble lifetime and energy against its maximum size are represented in Fig. S5.

While the exact solution to the Lambert W function in Eq. 3 of the main manuscript accurately captures the experiments, below we provide the approximation to help solve the equation with regular/popular functions. The following are the approximate relations:

$$\mathrm{W}_0(\psi) = \sum_{n=1}^{\infty} \frac{(-n)^{n-1}\psi^n}{n!}, \ |\psi| \le \frac{1}{e} \text{ and} \tag{S8}$$

$$\mathrm{W}_{-1}(\psi) \approx \log(-\psi) - \log(-\log(-\psi)) + \log(-\log(-\psi))/\log(-\psi)... \tag{S9}$$

Where $\psi = -e^{-(1+\widehat{X}_{\mathrm{max}})}$. Figure S6 shows the solution to the above equations for n upto 3 with

$$\widehat{t}_{\mathrm{osc}} = \mathrm{W}_0(\psi) - \mathrm{W}_{-1}(\psi). \tag{S10}$$

SII. LASER ENERGY ABSORBED BY THE LIQUID AND THRESHOLD ENERGY FOR BUBBLE FORMATION

The channel's geometry, such as, wall thickness, material, cross-section shape and dimension can influence the laser energy available for the liquid to absorb. For energy calibration, the energy transmitted through the channel (E_{trans}) was measured. We define an energy ratio, ϵ_{geom}, estimated by measuring the energy difference without the channels and channels filled with water. Therefore, if E_{supp} is the energy supplied to the channel, then for channels filled with water, $E_{\mathrm{trans}} = \epsilon_{\mathrm{geom}} E_{\mathrm{supp}}$. The channels filled with water was used as reference to measure energy absorbed in experiments since water is transparent to 532 nm wavelength and matches the refractive index of the aqueous dye (referred to as liquid).

For experiments with the aqueous dye, a portion or a whole the energy, $\epsilon_{\mathrm{geom}} E_{\mathrm{supp}}$, will be absorbed based on the absorption coefficient of the liquid and the distance the light will travel through the liquid. Therefore the energy transmitted through the channel with the liquid is, $E_{\mathrm{trans}} = \epsilon_{\mathrm{L}}\epsilon_{\mathrm{geom}} E_{\mathrm{supp}}$, where ϵ_{L} is the transmission ratio of the liquid. Therefore using the Beer–Lambert law [2], the absorbance (A_{abs}) of the liquid can be calculated as,

$$A_{\mathrm{abs}} = -\log_{10}(\epsilon_{\mathrm{L}}) = -\log_{10}(E_{\mathrm{trans}}/(\epsilon_{\mathrm{geom}} E_{\mathrm{supp}})). \tag{S11}$$

Furthermore, the absorbance can be written as, $A_{\mathrm{abs}} = \alpha\,d_{\mathrm{L}}$, where α is the absorption coefficient and d_{L} is the distance the light will travel through the liquid. d_{L} is equal to the hydraulic diameter (d_{h}) for circular and square channels. For rectangles it is the shorter edge length of the cross-section. Since the red dye (RD81, Sigma-Aldrich) concentration in the liquid is consistent through this work (0.5 wt%), therefore α is a constant. The α of the liquid is estimated experimentally by measuring the transmitted light as, $\alpha = A_{\mathrm{abs}}/d_{\mathrm{L}}$. Figure S7 shows the estimation of α from experiments using the A_{abs} against d_{L} line slope.

For $r \in [-d_\mathrm{L}/2, d_\mathrm{L}/2]$ is the radial position from the channel's geometric centre, the laser energy absorbed at the laser focal spot is required to estimate the threshold energy for bubble formation. An estimation for the transmitted energy as a function of r can be represented as, $E_\mathrm{trans} = 10^{-\alpha(d_\mathrm{L}/2+r)}\epsilon_\mathrm{geom}E_\mathrm{supp}$. By further differentiating it, we get,

$$\mathrm{d}E_\mathrm{trans} = -\epsilon_\mathrm{geom}E_\mathrm{supp}\alpha\log(10)10^{-\alpha(d_\mathrm{L}/2+r)}\,\mathrm{d}r. \tag{S12}$$

In the above expression for $r > d_\mathrm{L}/2$ is where the energy meter is positioned to calibrate the transmitted energy. Thus $r = 0$ correspond to the position of the laser spot, and $\mathrm{d}E_\mathrm{trans}(r = 0)$ is the energy absorbed at the laser spot. This absorbed energy will be used for sensible and latent heat of the liquid, resulting in the energy balance, $\mathrm{d}E_\mathrm{trans}(r = 0) = -\pi w_0^2\rho_\mathrm{L}(c_p\Delta T + H_\mathrm{L})\,\mathrm{d}r$. Where $2w_0$ is the laser spot diameter, c_p is the specific heat, ΔT is the rise in temperature and H_L the latent heat of vaporization. Thus the energy at the laser spot can be expressed as,

$$\epsilon_\mathrm{geom}E_\mathrm{supp} = \frac{\pi w_0^2\rho_\mathrm{L}(c_p\Delta T + H_\mathrm{L})}{\alpha\log(10)10^{-\alpha(d_\mathrm{L}/2)}}. \tag{S13}$$

In other words, the total energy absorbed by the liquid, E_abs, can be theorized as follows,

$$E_\mathrm{abs} = (1-\epsilon_\mathrm{L})\epsilon_\mathrm{geom}E_\mathrm{supp} = (1-10^{-\alpha d_\mathrm{L}})\frac{\pi w_0^2\rho_\mathrm{L}(c_p\Delta T + H_\mathrm{L})}{\alpha\log(10)10^{-\alpha(d_\mathrm{L}/2)}}. \tag{S14}$$

In the above theoretical expression for the absorbed laser energy, by substituting the values of properties of the liquid we can theoretically calculate the threshold energy for bubble formation (E_th). For a bubble to form, we use the spinodal temperature of water as the necessary condition - temperature at which the water explosively turns into vapor. Thus as the water reaches the spinodal temperature (577 K) [3], $E_\mathrm{th} = E_\mathrm{abs}$. Since the laser pulse width is 4 ns, the energy from the laser is transferred to the liquid within a short amount of time, which in turn vaporizes the liquid.

SIII. ANALYTICAL SOLUTION TO OSCILLATING FLOW WITH SINUSOIDAL PRESSURE GRADIENT

The equations discussed in this section are adapted from Wang [1].

Circular channels The velocity profiles within a channel with circular cross-section is as follows,

$$U(r,t) = \frac{(\Delta p/\Delta x)d_\mathrm{h}^2}{4\mu_e\sigma^2}[1 + c_1I_0(2\sigma r/d_\mathrm{h}) + c_2K_0(2\sigma r/d_\mathrm{h})]\,\exp(i\omega t), \tag{S15}$$

where, $\sigma = \sqrt{k + i\widehat{\omega}}$ and

$$c_1 = \frac{K_0(-\sigma) - K_0(\sigma)}{I_0(-\sigma)K_0(\sigma) - I_0(\sigma)K_0(-\sigma)},\ c_2 = \frac{I_0(\sigma) - I_0(-\sigma)}{I_0(-\sigma)K_0(\sigma) - I_0(\sigma)K_0(-\sigma)}. \tag{S16}$$

The I_n and K_n are the modified Bessel functions, and $\Delta p/\Delta x = p_\infty/l_\mathrm{L}$ is the pressure gradient along channel axis. Thus the flow is governed by two parameters, a non-dimensional frequency, $\widehat{\omega} = \rho_\mathrm{L}d_\mathrm{h}^2\omega/(4\phi\mu_e)$, and a porous medium factor, $k = \mu/(\mu_e D)$. D is the Darcy number, μ_e is the effective viscosity and ϕ the channel porosity. For pure fluid flows, $D \to \infty$, $\mu = \mu_e$ and $\phi = 1$ [4]. Thus the transient mean flow velocity is,

$$U(t) = \frac{4}{\pi d_\mathrm{h}^2}\int_0^{d_\mathrm{h}/2} U(r,t)\,2\pi r\mathrm{d}r = \frac{2\,(\Delta p/\Delta x)}{\mu_e\sigma^2}\,\mathbb{Re}\Big\{[0.5 + c_1I_1(\sigma)/\sigma]\,\exp(i\omega t)\Big\}, \tag{S17}$$

where $\mathbb{Re}$ is the real part of a complex number.

Figure S8A shows the predicted velocity profiles for an oscillating flow with pressure gradient $p_\infty\cos(\omega t)/l_\mathrm{L}$. The representative solution is for $d_\mathrm{h} = 300\,\mu\mathrm{m}$, $L = 50\,\mathrm{mm}$ and $t_\mathrm{osc} = 892\,\mu\mathrm{s}$. Figure S8B illustrates the velocity profiles along the channel's radial direction.

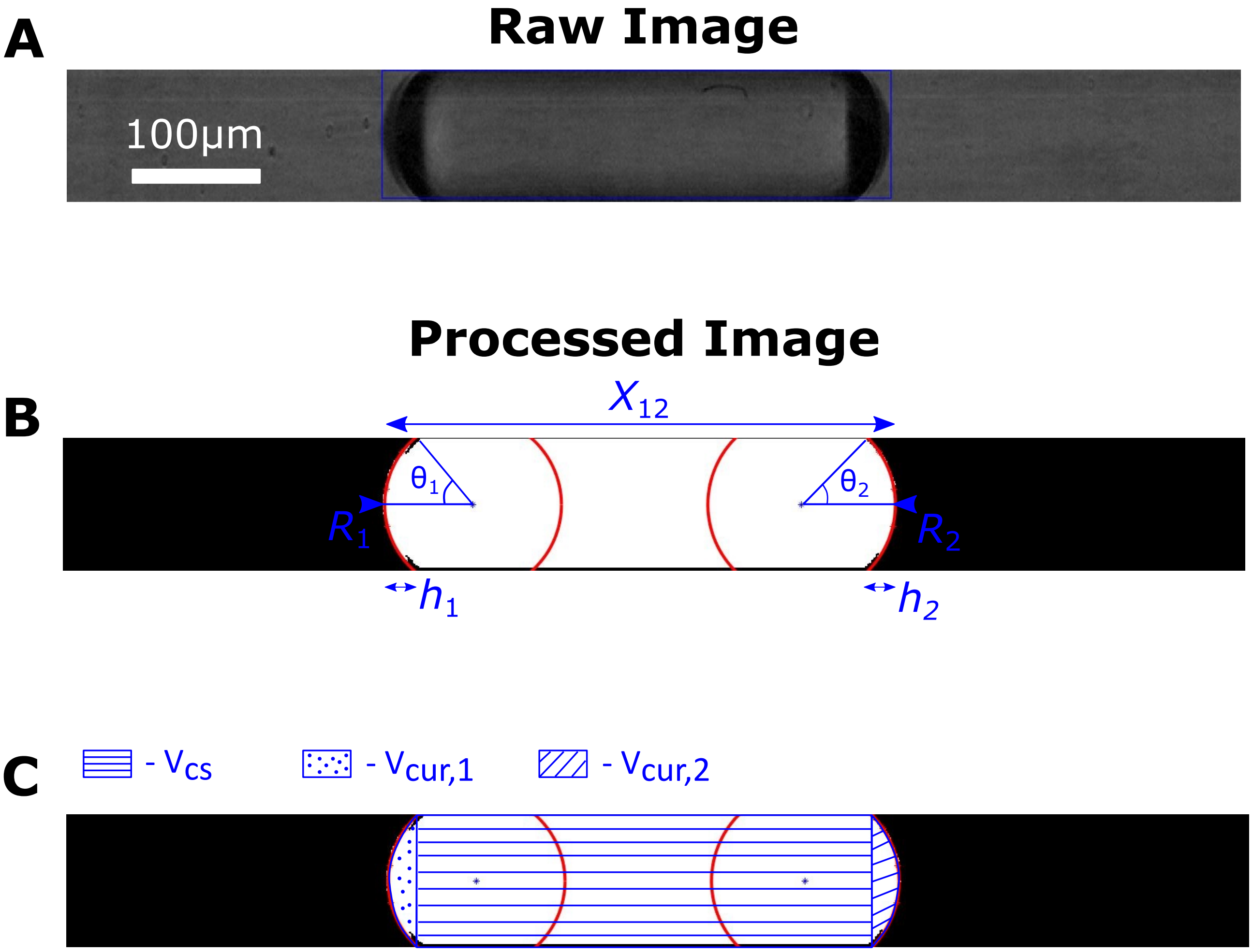

FIG. S1. (A) Raw image from experiments. (B) Processed image. X_{12} is the distance between the extreme bubble ends. R_1 and R_2 are the radius of curvature for the left and right ends respectively. h_1 and h_2 are the width of the curvature for the left and right ends respectively. (C) V_{cs} is the cross-section volume and V_{cur} is the curvature volume. The total volume of the vapor bubble, $V_{\mathrm{b}} = V_{\mathrm{cs}} + 2V_{\mathrm{cur}}$.

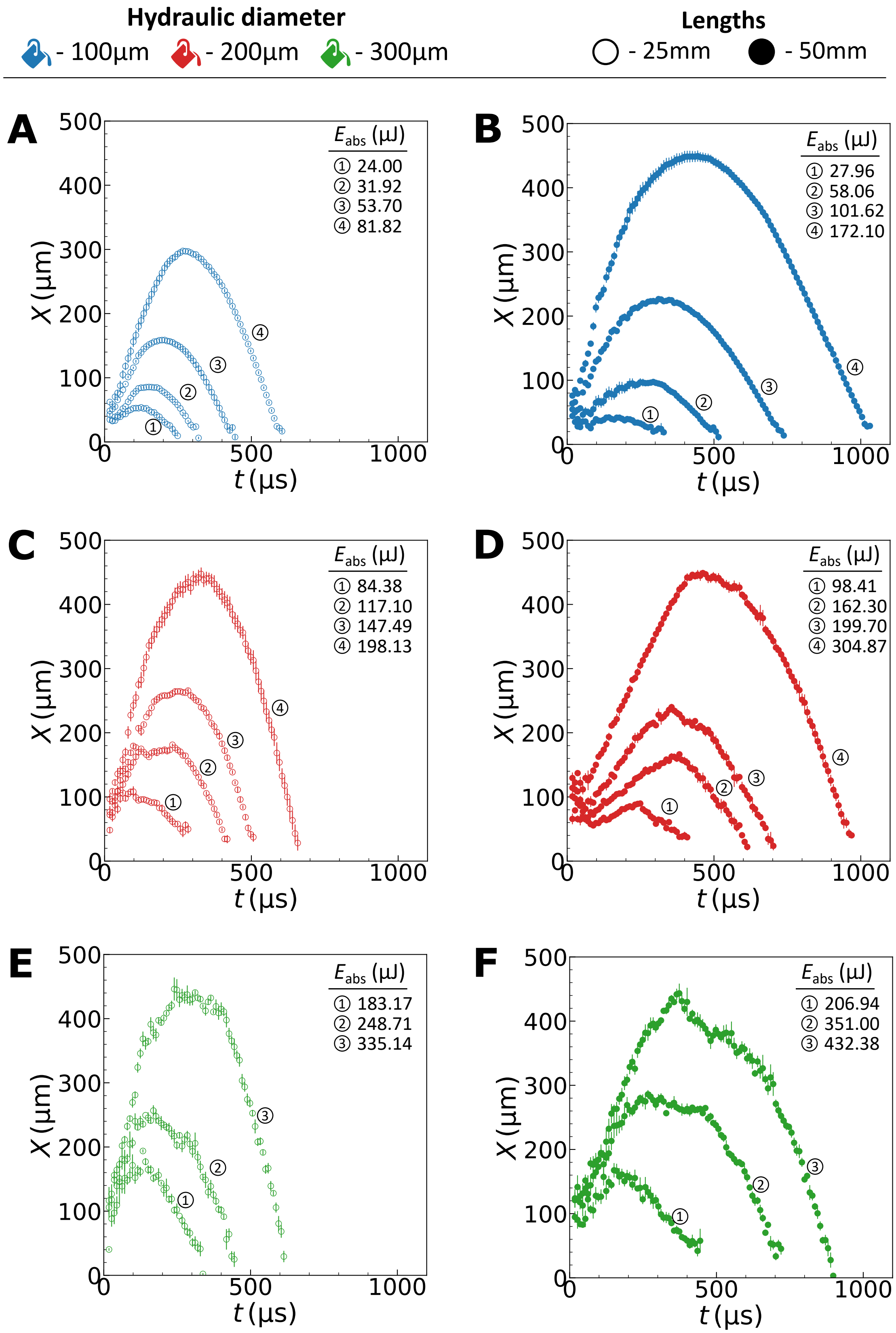

FIG. S2. (A-F) Bubble size (X) against time (t) for a channel with circular cross-section. The colors represent the dimension; the markerfacecolors represent the channel length. The error bars represent the standard error over 5 trials.

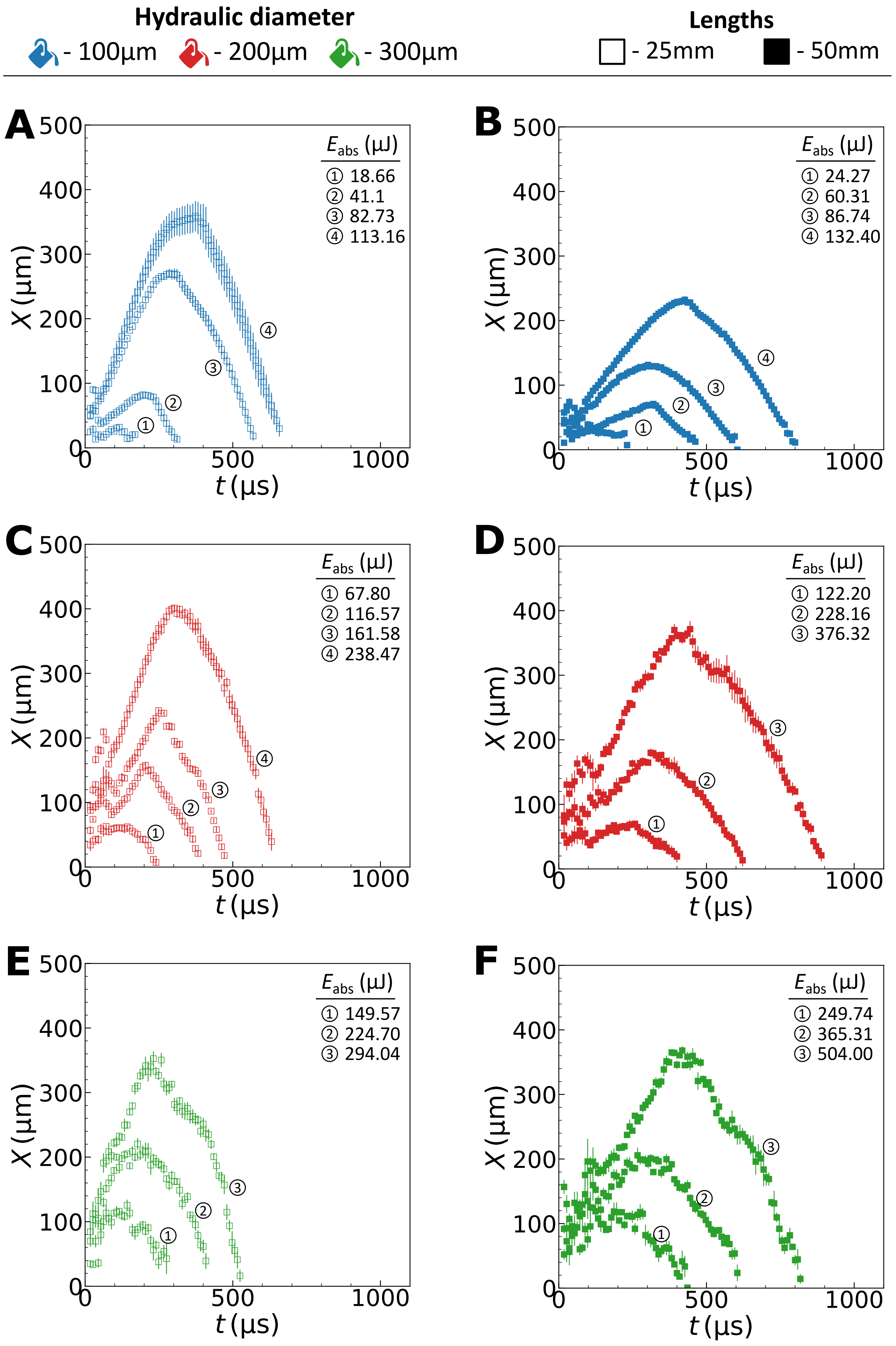

FIG. S3. (A-F) Bubble size (X) against time (t) for a channel with square cross-section. The colors represent the dimension; the markerfacecolors represent the channel length. The error bars represent the standard error over 5 trials.

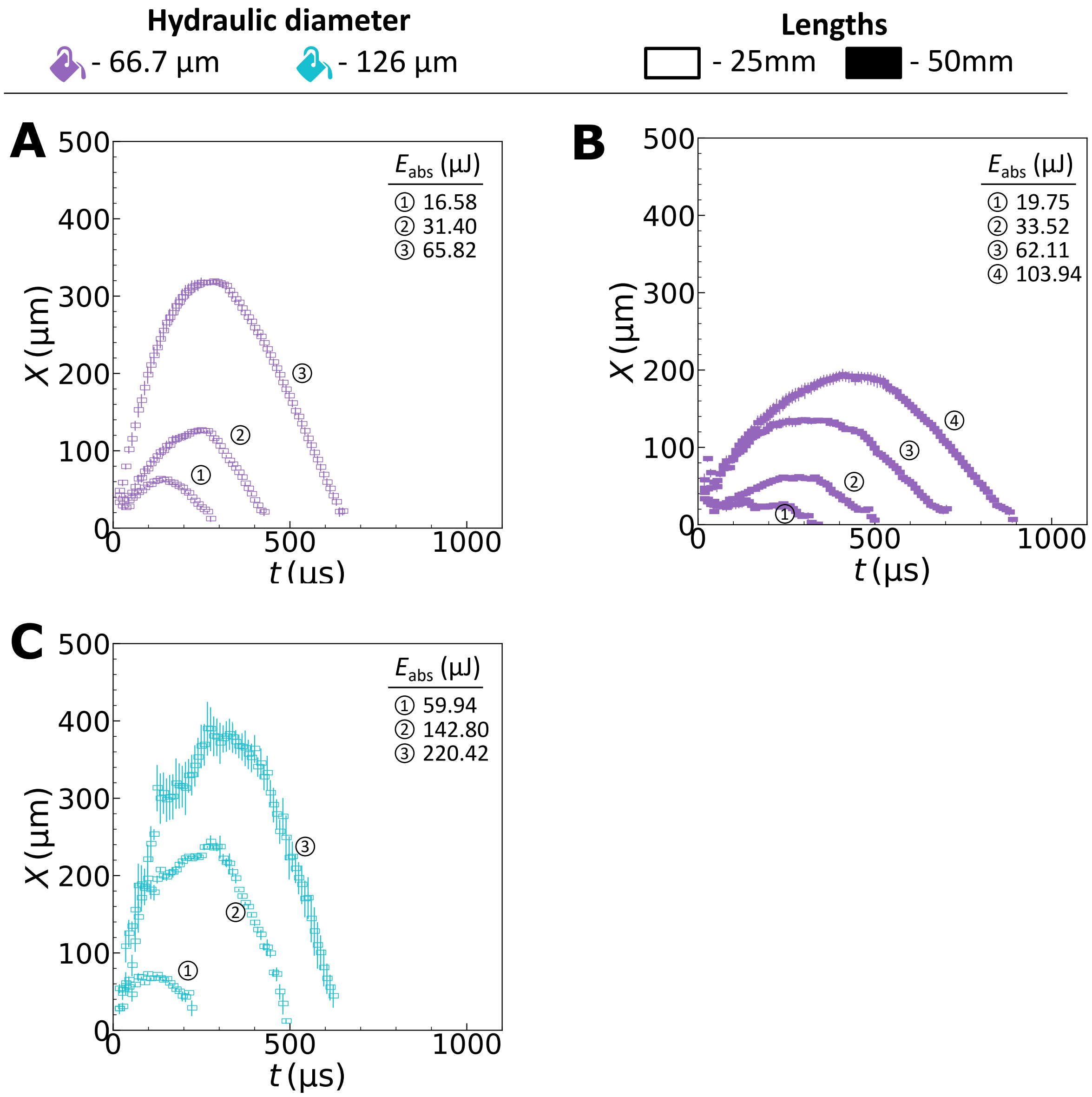

FIG. S4. (A-C) Bubble size (X) against time (t) for a channel with rectangular cross-section. The colors represent the dimension; the markerfacecolors represent the channel length. The error bars represent the standard error over 5 trials.

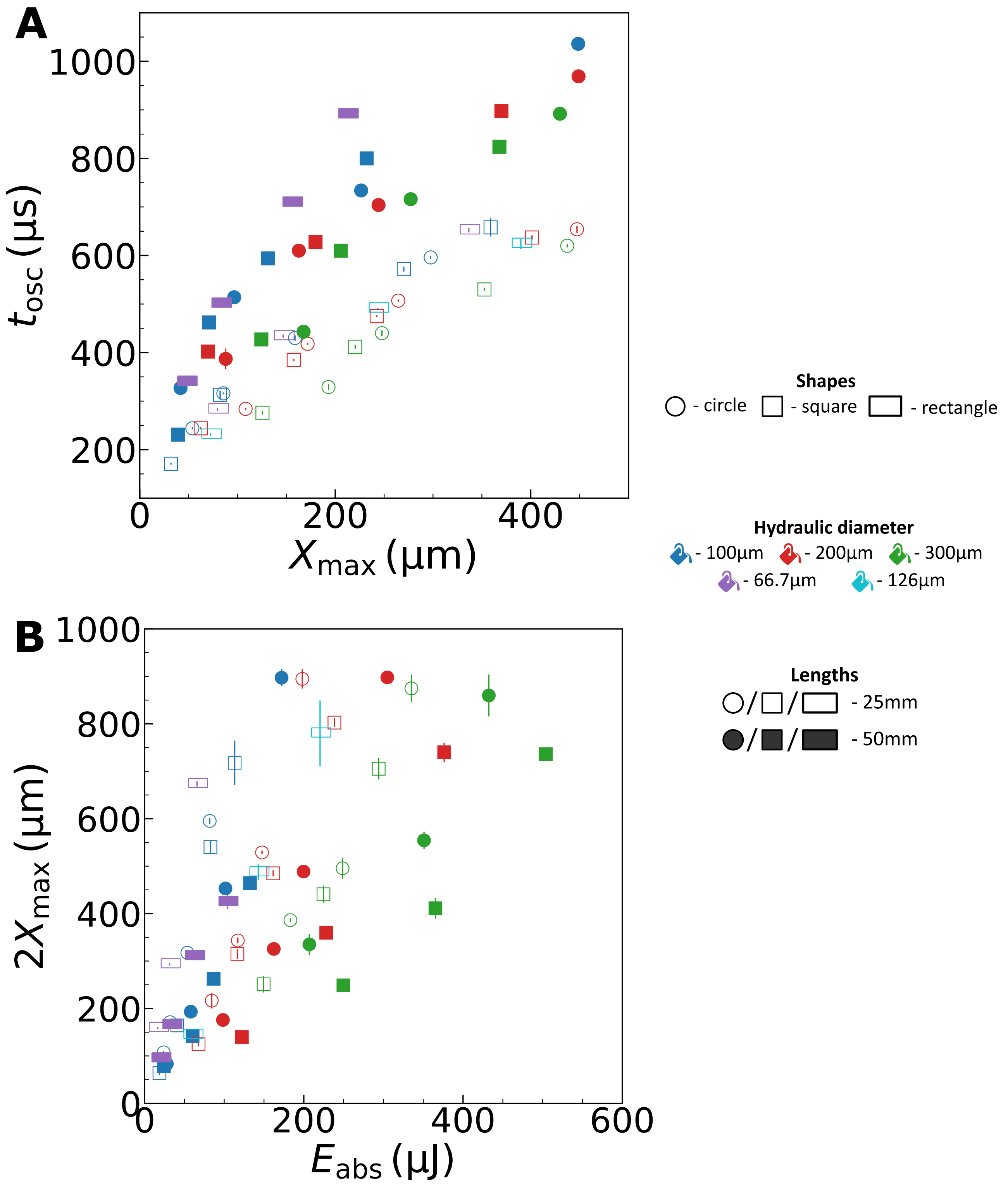

FIG. S5. (A) The lifetime ($t_{\rm osc}$) against maximum size ($X_{\rm max}$) of the bubble. (B) The maximum bubble size ($X_{\rm max}$) against the laser energy absorbed by the liquid ($E_{\rm abs}$). The marker shapes represent the shape of the cross-section; the colors represent the dimension; the markerfacecolors represent the channel length. The error bars represent the standard error over 5 trials.

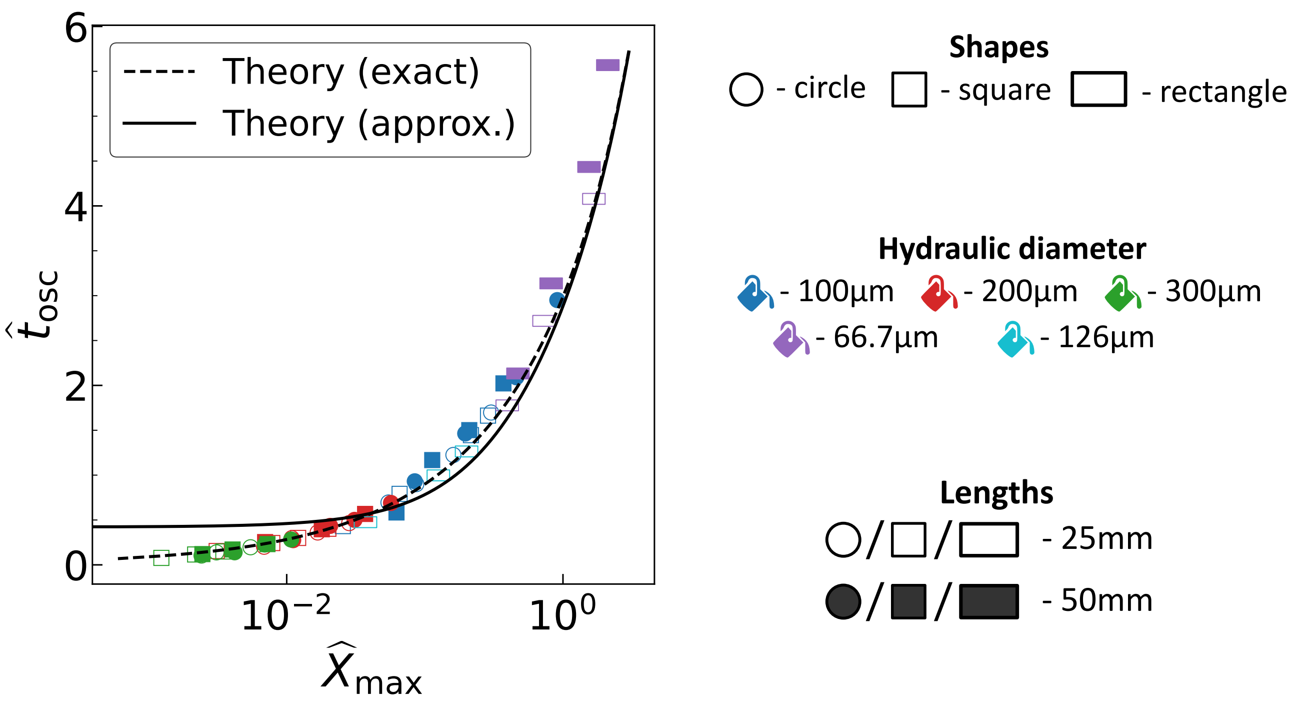

FIG. S6. The approximate solution to Eq. S10 employing Eqs. S8 and S9 is represented using the continuous line, while the exact solution to Eq. S10 is represented using dashed line. The approximate solution is with n upto 3 in Eq. S8. The markers are the experiments performed.

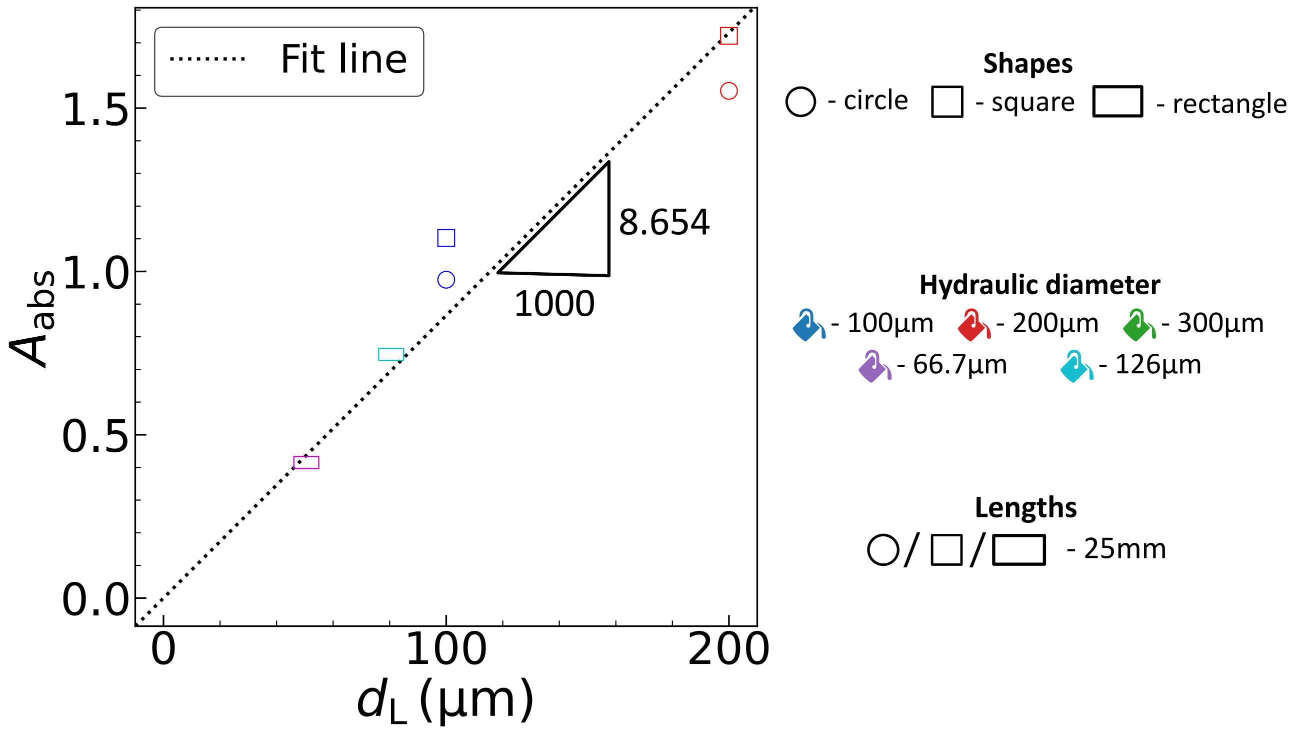

FIG. S7. The absorbance of the liquid ($A_{\rm abs}$) against the distance the light travels through the liquid ($d_{\rm L}$). The slope represents the absorption coefficient. The marker shapes represent the shape of the cross-section and the colors represent the dimension.

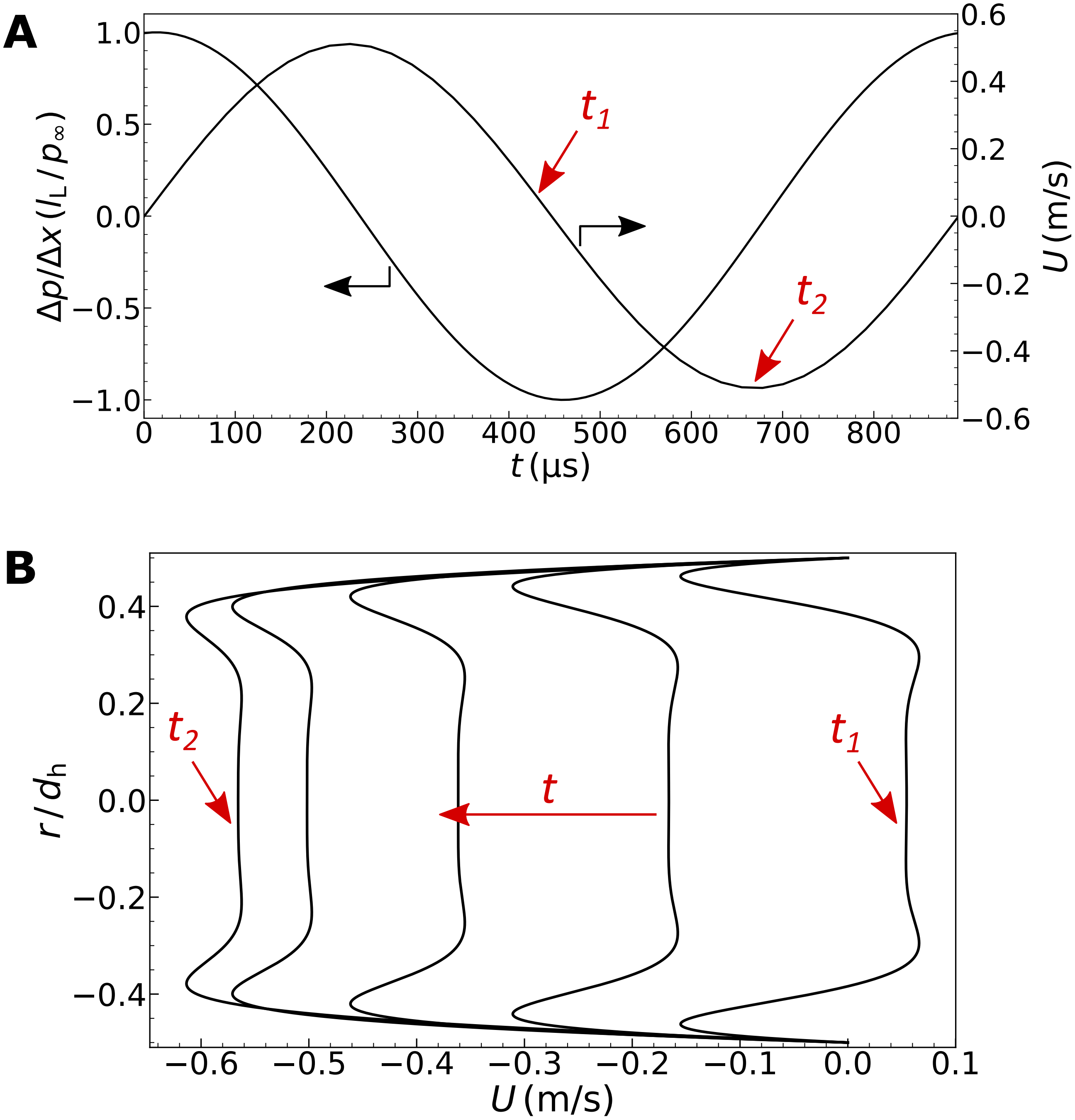

FIG. S8. (A) This representative analytical solution is for d_h = 300 µm, L = 50 mm and t_osc = 892 µs. U is the mean flow velocity. (B) The corresponding velocity profiles along the channel's radial direction (r) for the solution from (A). t_1 and t_2 are chosen time window to represent the flow. The direction of t represents the progress in time. The gradient in the velocity at the channel walls suggest a deceleration of the flow following the mean flow reversal (at the start of bubble collapse).

* h.b.eral@tudelft.nl

[1] C. Y. Wang, Transport in Porous Media **112**, 409 (2016).
[2] D. F. Swinehart, Journal of chemical education **39**, 333 (1962).
[3] R. Thiéry and L. Mercury, Journal of Solution Chemistry **38**, 893 (2009).
[4] W.-P. Breugem, Physics of Fluids **19**, 103104 (2007).
